# Enabling Allogeneic T Cell-Based Therapies: Scalable Stirred-Tank Bioreactor Mediated Manufacturing

**DOI:** 10.3389/fmedt.2022.850565

**Published:** 2022-05-30

**Authors:** Himavanth Gatla, Nicholas Uth, Yonatan Levinson, Ali Navaei, Alex Sargent, Senthil Ramaswamy, Inbar Friedrich Ben-Nun

**Affiliations:** Cell and Gene Therapy Research and Development, Lonza Inc., Rockville, MD, United States

**Keywords:** T cell manufacturing, bioreactor, allogeneic T cells, automation, closed process

## Abstract

Allogeneic T cells are key immune therapeutic cells to fight cancer and other clinical indications. High T cell dose per patient and increasing patient numbers result in clinical demand for a large number of allogeneic T cells. This necessitates a manufacturing platform that can be scaled up while retaining cell quality. Here we present a closed and scalable platform for T cell manufacturing to meet clinical demand. Upstream manufacturing steps of T cell activation and expansion are done in-vessel, in a stirred-tank bioreactor. T cell selection, which is necessary for CAR-T-based therapy, is done in the bioreactor itself, thus maintaining optimal culture conditions through the selection step. Platform's attributes of automation and performing the steps of T cell activation, expansion, and selection in-vessel, greatly contribute to enhancing process control, cell quality, and to the reduction of manual labor and contamination risk. In addition, the viability of integrating a closed, automated, downstream process of cell concentration, is demonstrated. The presented T cell manufacturing platform has scale-up capabilities while preserving key factors of cell quality and process control.

## Introduction

Chimeric antigen receptor (CAR) T cells are genetically engineered T cells expressing CAR, which is a non-major histocompatibility complex (MHC) restricted receptor containing a single-chain variable fragment (scFv) derived from an antibody, a transmembrane domain, and an intracellular signaling domain. Adoptive cell therapy using CAR T cells showed remarkable tumor specificity and robust anti-tumor immune responses, resulting in complete responses (1). To date, the Food and Drug Administration (FDA) has approved four autologous CAR T cells therapy products: tisagenlecleucel for acute lymphoblastic leukemia Novartis, 2017, axicabtagene ciloleucel for large B cell lymphoma Gilead, 2017, brexucabtagene autoleucel for mantle cell lymphoma in 2020 and relapsed or refractory B-cell precursor acute lymphoblastic leukemia (ALL) in 2021 (Gilead), and lisocabtagene maraleucel for relapsed or refractory large B-cell lymphoma (Bristol Myers Squibb, 2021) (2). Even though autologous CAR T cell therapies show remarkable therapeutic efficacy, they suffer several limitations. Autologous CAR T cell therapy requires T cell harvest from the patients, followed by genetic modification to express CAR and expansion, which could take at least 2 weeks. During this process, the progression of aggressive tumors could be lethal. Because the expansion of T cells depends on the quality attributes of the input cells, the inability to optimize the quality of a patient's T cells could lead to poor expansion yield. Expansion of tumor-infiltrating leukocytes provides an attractive strategy as the lymphocytes are primed against multiple tumor-associated antigens. However, their expansion *ex-vivo* is not effective, because of their exhausted phenotype and limited replicative potential (3). In addition to the above, the prohibitive cost of the procedure presents a critical challenge for autologous cell therapies (4). In contrast, allogenic cell therapy utilizes T cells from a donor. Because the donor does not undergo chemotherapy, the immune cells are more capable of eliminating target antigen harboring cells (5). Allogeneic cell therapy could be available as an ‘off-the-shelf' product, addressing the challenges of autologous cell therapy. Since matching of HLA-A, -B and -DR could potentially negate graft vs. host disease (GVHD), cell therapy products generated from selected individuals could apply to a wider population (6). Recently, Fate Therapeutics has reported the safety of administering allogeneic NK cell therapy products in doses up to 3 x 10^8^ cells (7). Hence, cell banks could be generated from optimal T cell subpopulations of healthy individuals, decreasing the production cost, while increasing the applicability and effectiveness. Therefore, allogeneic cell therapy has the potential to break the limitations of autologous cellular therapy (8). Owing to the promise of allogeneic cellular therapy, clinical trials evaluating the efficacy of allogeneic, ‘off-the-shelf', CAR T cells, targeting various tumor-associated antigens, are in progress (9, 10). CTX110 (CRISPR Therapeutics) (11), UCART19 in clinical trials NCT02808442 and NCT02746952 (Cellectis), and PBCAR0191 (Precision Biosciences) (12) are to name a few. Preliminary results show that UCART19 when administered to treat adult and pediatric patients with high-risk CD19^+^ relapsed/refractory B-cell acute lymphoblastic leukemia showed an efficacy of 88% of patients achieving complete responses (13). Furthermore, PBCAR0191 has shown similar efficacy rates compared to autologous CAR T cell therapy (14). Given the progress in cellular therapy, especially on the allogeneic front, the requirement for industrial-scale production of T cell products is inevitable. According to the Leukemia and Lymphoma Society, in the USA the expected number of new cases per year for leukemia is ~60,000, and for lymphoma is ~90,000 (https://www.lls.org/facts-and-statistics/facts-and-statistics-overview#Leukemia). While the number of CAR T cells per dose varies, estimates suggest the requirement of 3 trillion CAR T cells/year to treat hematological malignancies and 150 trillion CAR T cells for solid tumor malignancies. To meet the required batch sizes, scaling up a static flask-, in which the cells are grown on a flat surface (2D) will result in added labor, lab footprint, variability, risk of contamination, and poor process control. Stirred tank bioreactors (STRs) are available from small scale (1 L), bench scale (1–10 L) to pilot scales (10–1,000 L). Because the scale-up transfer kinetics are well characterized for STRs, they provide an excellent option, meeting the demand (15). Furthermore, as STRs are closed, automated, and GMP co-based culture compatible systems with an in-process control, T cell manufacturing in STRs significantly decreases the labor, batch to batch variation, and contamination risk. T cells are non-adherent, inherently cultured in suspension as single cells. This makes suspension-based bioreactors suitable for their expansion, without the artificial need to adapt them to suspension culture conditions and culture them in aggregates or attached to carriers. Similar to other cell types, expansion of T cells are sensitive to the buildup of cell metabolites, such as ammonia and lactate, in the culture media and hence requires media replenishment (16). Even though fresh nutrients can be supplied using the fed-batch culture method, the buildup of inhibitory agents in the media could potentially inhibit the cell growth, warranting continuous media perfusion. The attributes that make T cells suitable for expansion in 3D suspension cultures (such as stirred tank bioreactors where the cells are grown in 3-dimensional suspension culture) also present a challenge for automated, continuous, media change. Because the diameter of T cells ranges from 5 to 10 mm, perfusion of T cell cultures has been difficult, with major responsible factors being frequent filter fouling and cell escape. Hence, a scale-up technology equipped with continuous media perfusion enabling T cell expansion is urgently needed.

Allogeneic CAR T cell generation requires genetic engineering, which involves knocking out T cell receptor (TCR), selective knockout of MHC I/II allele, on top of the genetic manipulation needed for the expression of the CAR molecule. Even though cutting edge gene-editing technologies, such as Zinc- finger nucleases (ZFNs) and MegaTALs, clustered regularly interspaced short palindromic repeats (CRISPR/Cas9), and transcription activator-like effector nuclease (TALEN), provide tools for successful deletion and insertion of desired genes, although 100% efficacy cannot be guaranteed, thus, resulting in the presence of unedited cells in the culture (17). The presence of TCR-positive cells in the final allogeneic cell therapy product could result in graft vs. host disease, which could be life-threatening. Hence, depletion of undesired cells is mandatory and requires a separate unit operation, which increases the chances of cell loss and contamination. To achieve the clinical demand of high cell numbers, scaling up the depletion step could potentially involve unreasonable processing time and/or non-viable scale-out.

Here, we describe a closed and scalable platform for T cell manufacturing. This platform offers in-process control and includes T cell expansion in a stirred tank bioreactor with continuous media perfusion. Following T cell expansion, undesired cell depletion is performed in-vessel, avoiding a unit operation, while reducing labor and contamination risks. Furthermore, cell processing and concentration are performed in a closed manner, without compromising cell yield and the characteristics of the cells.

## Materials and Methods

Here, we describe the methods utilized to isolate T cells from peripheral blood mononuclear cells (PBMCs), activate and expand in different culture conditions, process parameters used for T cell expansion, cell purification, concentration, and characterization.

### Isolation of T Cells From Peripheral Blood Mononuclear Cells (PBMCs)

Human peripheral blood mononuclear cells (PBMCs) (Lonza Cat #4W-270C) were thawed rapidly in a 37°C water bath until a small bit of ice was left in the vial. Thawed cells were added dropwise to EasySep^TM^ buffer (Stem Cell Technologies Cat #20144). The cells were centrifuged at 300 RCF for 5 min at room temperature (RT). The supernatant was discarded and the cells were reconstituted in 50 mL of EasySep^TM^ buffer. Cell concentration and viability were evaluated using the NucleoCounter NC-200 (Chemometec, Denmark). The cells were centrifuged again at 300 RCF for 5 min at RT. The supernatant was discarded and the cells were reconstituted at 50 x 10^6^ cells/mL in X-VIVO^TM^ 15 Serum-free Hematopoietic Cell Medium (Lonza Cat #04-418Q). A sample was aliquoted for immune-phenotype staining. T cells were isolated using a T cell isolation kit (Stem Cell Technologies Cat #17951) by following the manufacturer's protocol. Post-T cell isolation, cell concentration, and viability were evaluated using the NucleoCounter NC-200 (Chemometec, Denmark), and a sample was aliquoted for immune-phenotype staining.

### T Cell Activation and Expansion in Spinner Flasks

T cell complete media was prepared by adding Human AB serum (Sigma Cat #H4522) to a final concentration of 5% and recombinant human IL-2 (Peprotech Cat #200-02) to a final concentration of 50 ng/mL to X-VIVOT^M^ 15 (Lonza Cat #04-418Q). After T cell isolation (as described in 4.1), cells were seeded in spinner flasks at 1 x 10^6^ cells/mL in 40 mL of T cell complete media. Cells were stimulated with ImmunoCult^TM^ Human CD3/CD28 T-cell activator (Stem Cell Technologies Cat #10991) for 3 days following the manufacturer's instructions. Media was added on day 5 to adjust the cell density to 1 x 10^6^ cells/mL and media was doubled on day 8. Agitation in one condition of the spinner flasks was maintained at 75 RPM for the entirety of 11 days and agitation in the other was started at 50 RPM through days 1–4 followed by 100 RPM through days 5–11. Cell count and viability were determined by sampling on culture days 4, 7, 8, and 11.

### T Cell Activation and Expansion in G-Rex®

T cell complete media was prepared by adding Human AB serum (Sigma Cat #H4522) to a final concentration of 5% and recombinant human IL-2 (Peprotech Cat #200-02) to a final concentration of 50 ng/mL to X-VIVO^TM^ 15 (Lonza Cat #04-418Q). After T cell isolation (as described in 4.1), a 1L G-Rex® (Wilson Wolf Cat #G-Rex®100M-CS) was inoculated with T cells at 0.5 x 10^6^ cells/mL in 300 mL of T cell complete media. T cell activation was performed in the G-Rex® using ImmunoCult^TM^ Human CD3/CD28 T-cell activator (Stem Cell Technologies Cat #10991) for 3 days following the manufacturer's instructions. T cell culture in the G-Rex® was maintained at 37^o^C, 5% CO_2_ humidified atmosphere. After T cells were activated for 3 days, 700 mL of T cell complete media was added and was cultured until day 14.

### T Cell Activation and Expansion in a Stirred Tank Bioreactor

The BioBlu single-use bioreactor vessel was set up according to the manufacturer's instructions (Eppendorf, 1386000300). Briefly, the 1-L vessel was equipped with probes required for online monitoring (Mettler Toledo) of key parameters including the percentage of dissolved oxygen (DO), pH, and temperature. The bioreactor was controlled using a G3 Lab Universal controller (Thermo Fisher Scientific). T cell complete media was prepared by adding Human AB serum (Sigma Cat #H4522) to a final concentration of 5% and recombinant human IL-2 (Peprotech Cat #200-02) to a final concentration of 50 ng/mL to X-VIVO^TM^ 15 (Lonza Cat #04-418Q). Prior to inoculation, 400 mL of T cell complete media was introduced into the bioreactor and was equilibrated with air. After T cell isolation, the bioreactor was inoculated with 200 x 10^6^ T cells at a seeding density of 0.5 x 10^6^ cells/mL. T cell activation was performed in the bioreactor using ImmunoCult^TM^ Human CD3/CD28 T cell activator (Stem Cell Technologies Cat #10991) for 3 days following the manufacturer's instructions. T cell culture in the bioreactor was maintained at 37^o^C, 88 RPM agitation (except otherwise mentioned), and pH < 7.2. After T cells were activated for 3 days, 600 mL of T cell complete media was added. To determine cell growth and fold expansion, 15 mL samples were taken in triplicates at various time points along the run and the NucleoCounter NC-200 (Chemometec, Denmark) was used to measure the cell number and viability. When the viable cell density of T cells has reached 2.0 × 10^6^ cells/mL, perfusion with fresh T cell complete media was initiated at a rate of one vessel volume per day (VVD). On the indicated days, samples were used for immune-phenotypic analysis and single-cell secretome analysis. To monitor the changes in key metabolites, 5 mL samples were taken from the bioreactor at various time points along the run. Offline monitoring to determine changes in parameters such as pH and key nutrients was performed using the BioProfile FLEX Analyzer (Nova Biomedical).

### Continuous Media Perfusion

Cell retention and continuous media perfusion were performed using Xcell^TM^ Alternating tangential flow filtration (ATF) 2 single-use device (Repligen Cat #suATF2-S02PES). ATF was aseptically connected to the bioreactor on Day 0 and the ATF column was wetted using X-VIVO^TM^ 15 Serum-free Hematopoietic Cell Medium (Lonza Cat #04-418Q). On day 8, when the cells have reached 2.0 x 10^6^ cells/mL, perfusion was enabled at 0.5 liters per minute (LPM) ATF rate. Media in and media out were set to 0.7 mL/min, maintaining 1 VVD.

### CD4^+^ T Cell Depletion

After cell expansion, on day 14, perfusion was stopped and the required number of Dynabeads^TM^ CD4 (Thermo Fisher Scientific Cat #11145D) were washed in isolation buffer (DPBS supplemented with 0.1% human AB serum and 2 mM EDTA) as per the manufacturer's instructions. The beads were added to the bioreactor and were incubated for 30 min in agitation. Incubation with magnets was performed for the required time period. At indicated times, 10 mL of samples were drawn out of the bioreactor for taking pictures under 20x magnification of Rebel light microscope (Echo, USA), followed by calculation of residual bead concentration (see below), then, staining for immune-phenotypic analysis (see below), and evaluation of viable cell concentration and cell viability (NucleoCounter NC-200).

### Harvest of T Cells From Stirred Tank Bioreactor

T cells in the 1-L bioreactor were collected on day 14 of total cell culture. With the connection between the bioreactor and ATF closed, and in continuous agitation, the cell solution in the bioreactor was pumped into a sterile 1-L bag. The same 1-L bag was connected to the harvest line of the ATF and the cell solution present in the ATF was harvested. About 15 mL of cell solution in the harvest bag was sampled for immune-phenotypic analysis (see below), evaluation of viable cell concentration, and cell viability (NucleoCounter NC-200).

### Downstream Processing

A bag containing the T cell suspension harvested from the bioreactor was sampled in triplicate, and the viabilities and cell densities were then determined using a NucleoCounter NC-200. The average viable cell density (VCD) was used to calculate the concentrated volume that would be harvested by the kSep (Equation 1, see Equations). A kSep (Sartorius) was fitted with a 400.50 rotor, which functions as a 1/3.5 scale-down model for the kSep400. The associated 400.50 single-use kits (chamber set and valve set) were then installed. A solution of PlasmaLyte-A (Baxter) and (.25%) human AB serum (Sigma Cat #H4522) was used to prime the system. A static centrifugation speed of 1,000 g was used. The fluidized bed was established at a 24 mL/min flow rate for 60 min and was harvested at 120 mL/min into a harvest bag. For the entirety of the concentration process, 5 mL samples were drawn from the stream exiting the keep chamber and tested using the NucleoCounter NC-200 (Chemometec, Denmark) to monitor the number of cells escaping the fluidized bed. After 1 L of cell suspension was processed, the concentrated cells were harvested. The volume of the concentrate was verified, and samples were taken to determine viability and cell density. The remaining concentrate was cryopreserved.


(1) $$\begin{array}{l} Concentrated\;Vol=\frac{\left(Feed\;VCD\right)\;*\;\left(Feed\;Vol\right)}{Target\;Concentrated\;VCD} \end{array}$$

For concentration by ekko^TM^, an ekko^TM^ single-use cartridge was installed, and the chamber was primed with 100 mL of wash buffer (A solution of PlasmaLyte-A (Baxter) and (.25%) human AB serum (Sigma Cat #H4522). The feed was recirculated from an acoustic fluid bed at 120 W and a flow rate of 70 mL/min. After 1 L of cell suspension was processed, the concentrated cells were harvested in 2 cycles. The volume of the concentrate was verified, and samples were taken to determine viability and cell density. The remaining concentrate was cryopreserved.

### Cryopreservation

Human T cells were suspended in cryopreservation solution (CS10, Biolife Solutions Inc., 210102). Cryovials were cryopreserved by Cryomed™ Controlled-rated Freezer (Thermo Fisher Scientific, Model 7456) and subsequently stored in liquid nitrogen until use.

### Flow Cytometry

Quantitative detection of T cell differentiation, senescence, and exhaustion status was performed using flow cytometry. Briefly, 300,000 cells were live-stained for the cell surface markers: CD62L (Biolegend Cat #304806), CD45RA (Biolegend Cat #304108), CD45RO (Biolegend Cat #304218), CD3 (BD Biosciences Cat #564713), CD4 (CD Biosciences Cat #560158), CD8 (Biolegend Cat #301028), KLRG1 (Biolegend Cat #367716), CTLA4 (BD Biosciences Cat #563931), PD-1 (BD Biosciences Cat #564324), CD57 (Biolegend Cat #393304), and ZOMBIE 421 (Biolegend Cat #423114). The samples were processed using FACS Celesta^TM^ (Becton Dickinson), and data were acquired using the BD FACS Diva Software followed by analysis using FlowJo v10 software (FlowJo).

### Residual Bead Percentage

From each sample, 1 ml was dispensed into each of the 3 tubes. The tubes were centrifuged at 700 RCF for 5 min at room temperature. The cell pellet was resuspended in 1 mL of Dulbecco's phosphate-buffered saline (DPBS). To the cell solution, 4 mL of lysis buffer (1:1 ratio of DPBS and sodium hypochlorite) was added, mixed, and left at room temperature for 5 min. The tubes were centrifuged at 700 RCF for 5 min at room temperature and 4.95 mL of supernatant was removed. The remaining 50 μL was mixed well and 10 μL was dispensed into each side of the hemocytometer. The number of beads in 4 corner squares was counted. The counting was repeated for the 2nd side of the hemocytometer. The remaining solution in the tube was mixed well and the counting was performed till the solution in the tube was completely counted. The residual bead count was calculated using Equation 2 (see Equations). Residual bead percentage concerning the viable cell density of the sample was calculated using Equation 3 (see Equations).


(2) $$\begin{array}{ll} Number\;of\;beads\;per\;ml= & \frac{\left(\text{sum of number of beads from each count}\right)\;*\;\left(100\right)}{4} \end{array}$$

(3) $$\begin{array}{ll} Residual\;bead\;percentage= & \frac{\left(100\right)\;*\;\left(residual\;beads\;per\;ml\right)}{\;VCD\;\left(\frac{cells}{ml}\right)} \end{array}$$

### T Cell Polyfunctionality Analysis Using Isoplexis

During the bioreactor run, 15 ml of samples were drawn for a multitude of analyses. At indicated time points, 1 mL of solution was centrifuged at 500 RCF for 5 min at room temperature. The supernatant was transferred into a new sterile 1.5-mL sterile tube and the pellet was cryopreserved as described in section 4.7. The cells were thawed as described in 4.1 and were recovered overnight at 5% CO_2_ and 37^o^C in T cell complete media supplemented with 10 ng/mL of human recombinant IL-2. CD4^+^ and CD8^+^ T cell enrichment were performed as described in 4.13. The two distinctive T cell populations were stimulated for 5 hours in 50 ng/mL phorbol 12-myristate 13-acetate (PMA) (Sigma-Aldrich Cat #P8139) and 1 mg/mL Ionomycin (Sigma-Aldrich Cat #I0634). The cells were loaded into the single-cell secretome barcode chip (Isoplexis Cat #PANEL-1001-8) for single-cell secretomes evaluation. A single cell functional profile was determined for each T cell type. Profiles were categorized into effector (Granzyme B, IFN-g, MIP-1a, Perforin, TNF-a, TNF-b), stimulatory (GM-CSF, IL-2, IL-5, IL-7, IL-8, IL-9, IL-12, IL-15, and IL-21), regulatory (IL-4, IL-10, IL-13, IL-22, TGF-b1, sCD137, and sCD40L), chemo attractive (CCL-11, IP-10, MIP-1b, and RANTES), and inflammatory (IL-1b, IL-6, IL-17A, IL-17F, MCP-1, and MCP-4) groups.

### Isolation of CD4^+^ and CD8^+^ T Cells

The CD4^+^ and CD8^+^ T cells were isolated from CD3^+^ T cells using CD4^+^ microbeads, human (Miltenyi Biotec Cat #130-045-101), and CD8^+^ microbeads, human (Miltenyi Biotec Cat #130-045-201). Briefly, the cells were labeled with specific microspheres and were passed through MACS LS columns (Miltenyi Biotec Cat #130-042-401) placed on the midiMACS Separator (Miltenyi Biotec Cat #130-042-302) and MACS Multistand (Miltenyi Biotec Cat #130-042-303). Enriched cells trapped in the column were plunged into a fresh collection tube, washed, and resuspended in T cell complete media. Samples were collected before and after isolation for flow cytometric analysis as described above.

## Results

### T Cells Expand Better in Agitation, Compared to Static Culture

**Figure 1:**
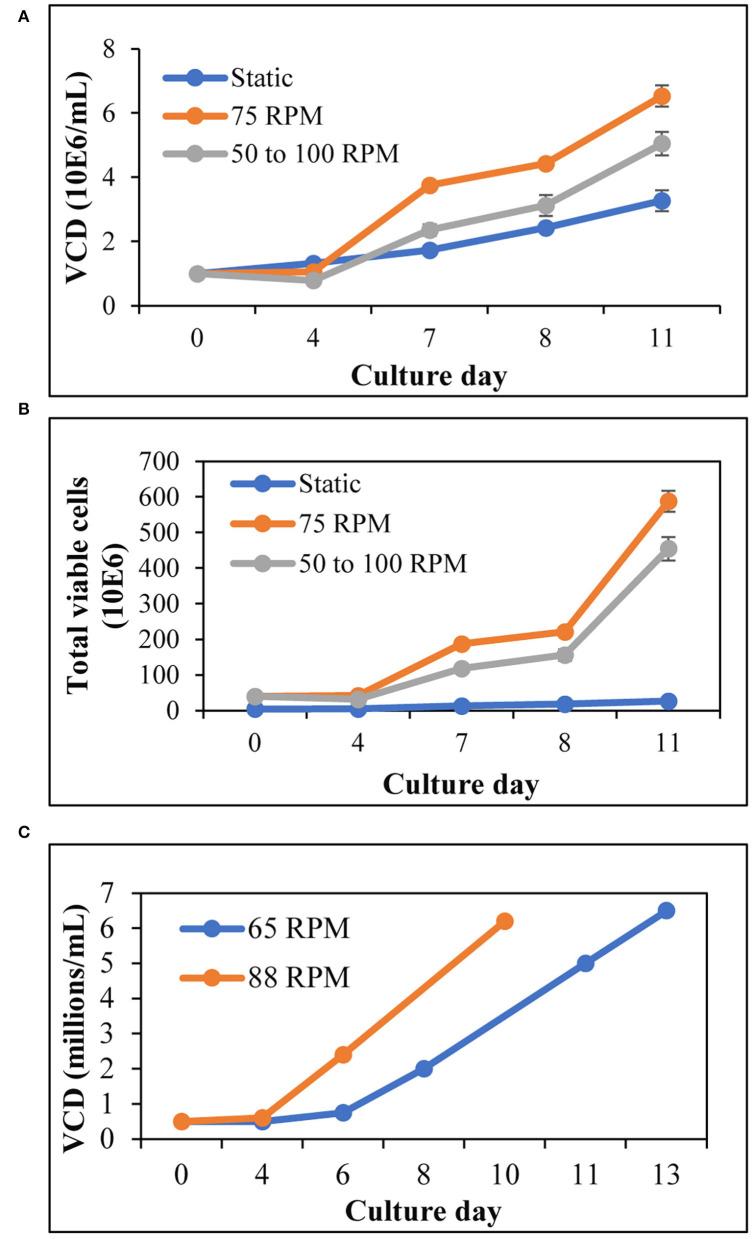
T cells expand better in agitation, compared to 2D flasks: **(A)** Viable cell density of T cells for 11 days expansion in 125-mL spinner flasks and T25 (2D) flask. The agitation was increased from 50 to 100 RPM on day 5; **(B)** Total number of T cells during expansion in 125 mL spinner flasks and T25 (2D) flask, as evaluated on specified days till day 11; **(C)** VCD of T cells expanded in 1-L STR at different agitation rates. The values represent mean ± S.E. of three experiments.

Stirred tank bioreactors utilize agitation as a mechanism to ensure the even distribution of gasses and nutrients. To determine whether T cells could be expanded in agitation, we have activated and cultured T cells at different agitation rates in spinner flasks and compared them to T cell expansion in T25 (2D) static flask. Cell counts performed at various time points throughout the run indicated that the viable cell density (VCD) and the total number of T cells in T25 (2D) static culture were consistently lower than in agitated conditions (Figures 1A,B). To test if a lower agitation rate during the activation, followed by increased agitation results in better T cell expansion when compared to a constant agitation rate, we have evaluated the VCD and total T cell expansion when the agitation is started at 50 RPM and increased to 100 RPM in comparison to agitation at a constant 75 RPM. Agitation at a constant 75 RPM resulted in higher VCD of T cells, compared to the VCD when agitation is initiated at 50 RPM and increased to 100 RPM on day 5 (Figure 1A). Furthermore, agitation at 75 RPM resulted in a 15-fold T cell expansion, compared to 10-fold T cell expansion observed when agitation is initiated at 50 RPM and increased to 100 RPM (Figure 1B). Maintaining the fed-batch media change regime and the range of the tip speed tested in the spinner flask, the expansion of T cells in a 1-L stirred tank bioreactor (STR) was evaluated at two different agitation rates. As shown in Figure 1C, agitation at 88 RPM resulted in a higher viable cell density of T cells, compared to agitation at 65 RPM.

### T Cell Activation in a Stirred-Tank Bioreactor and Continuous Cell Culture Media Perfusion Enable Steady Expansion

**Figure 2:**
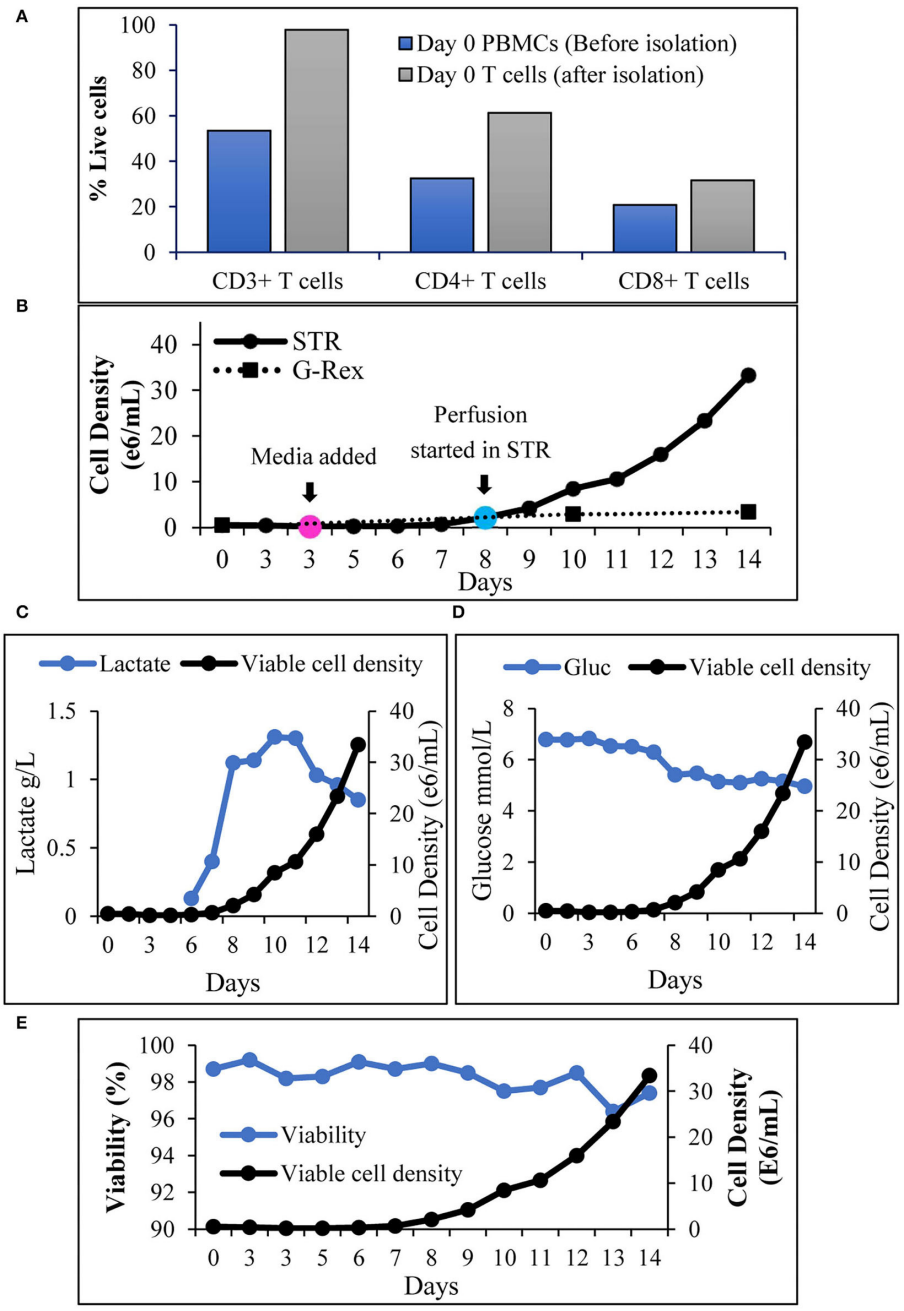
Alternating tangential flow mediated continuous perfusion in stirred tank bioreactor enables T cell expansion: **(A)** Prominent subsets of T cells before and after isolation from PBMCs. The values represent an average of 50,000 single cells per condition; **(B)** Viable cell density of T cells during 14 days expansion. Cell count in STR was performed at inoculation (day 0), before and after media addition (Day 3) and then each day till day 14. Cell count in G-Rex® was performed on day 0 at inoculation, on days 10 and 14. The values represent mean ± S.E. of three technical replicates; **(C)** Lactate levels during 14 days of T cell cultivation in the stirred tank bioreactor in comparison to viable cell density; **(D)** Glucose levels during 14 days of T cell cultivation in the stirred tank bioreactor in comparison to viable cell density; **(E)** Cell viability of T cells during 14 days of T cell cultivation in the stirred tank bioreactor. The values represent mean ± S.E. of three technical replicates.

An increase in cell density is accompanied by nutrient depletion and accumulation of inhibitory metabolites. Continuous media perfusion replaces the spent media with fresh media and provides optimal culture conditions, enabling cell expansion. To evaluate if continuous media perfusion can be performed using alternating tangential flow (ATF) without cell loss, we have tested the effect of 24 hr media perfusion by ATF on T cells, inoculated at 3 x 10^6^ cells/mL in 2L culture media in a 3L STR. As shown in Table 1, we did not observe a notable decrease in the VCD of the T cells, 24 h after perfusion. This was accompanied by the absence of T cells in the waste bag, indicating that ATF-mediated media change did not result in cell loss. Furthermore, evaluation of the weight of the waste bag 24 h after perfusion showed that continuous media perfusion of 1 vessel volume per day (1 VVD) was achieved without filter fouling. To evaluate if T cell expansion can be achieved in STR with continuous media perfusion, CD3^+^ T cells were isolated from peripheral blood mononuclear cells (PBMCs), inoculated into the stirred tank bioreactor, and were activated as described in materials and methods. Phenotypic evaluation of the cells after isolation showed that 98% of the cells were CD3^+^ (Figure 2A) with viability of >98% (data not shown). Post inoculation and activation in STR, expansion of T cells over 14 days were monitored, showing T cell VCD reaching 2 x 10^6^ cells/mL on day 8 (Figure 2B). With the increase in cell density, a concurrent increase in lactate levels was observed (Figure 2C). Cell retention and media exchange using ATF at 1 VVD curtailed lactate buildup and enabled the viable cell density to reach 33.5 x 10^6^ cells/mL on day 14 (Figure 2C). In comparison, T cells isolated from the same donor and cultured in static mode, in G-Rex®, resulted in a VCD of 3.4 x 10^6^ cells/mL on day 14 (Figure 2B). In addition to avoiding the lactate accumulation, ATF-mediated perfusion at 1 VVD enabled nutrient replenishment, as shown by stable glucose levels during cell expansion in Figure 2D. Furthermore, agitation in the stirred tank bioreactor in combination with ATF mediated cell movement did not lead to cell death, demonstrated via stable >96% cell viability (Figure 2E).

**Table 1:** Evaluation of alternating tangential flow filtration (ATF) for continuous media perfusion and cell retention.

**Variable**	**Before perfusion**	**24 h after perfusion**
Cell concentration (cells/mL)	3 × 10^6^	2.5 × 10^6^
Cell viability (percentage)	94	96.2
Cell concentration in waste	0	0
(cells/mL)		
Volume in media bag (L)	2	0
Volume in waste bag (L)	0	2

*Cell concentration, viability, and cell presence in waste bag and volume in and out of bioreactor were evaluated*.

### T Cell Activation in a Stirred-Tank Bioreactor With Continuous Perfusion Results in T Cells With Stemness Over Exhaustion and Senescence Markers

**Figure 3:**
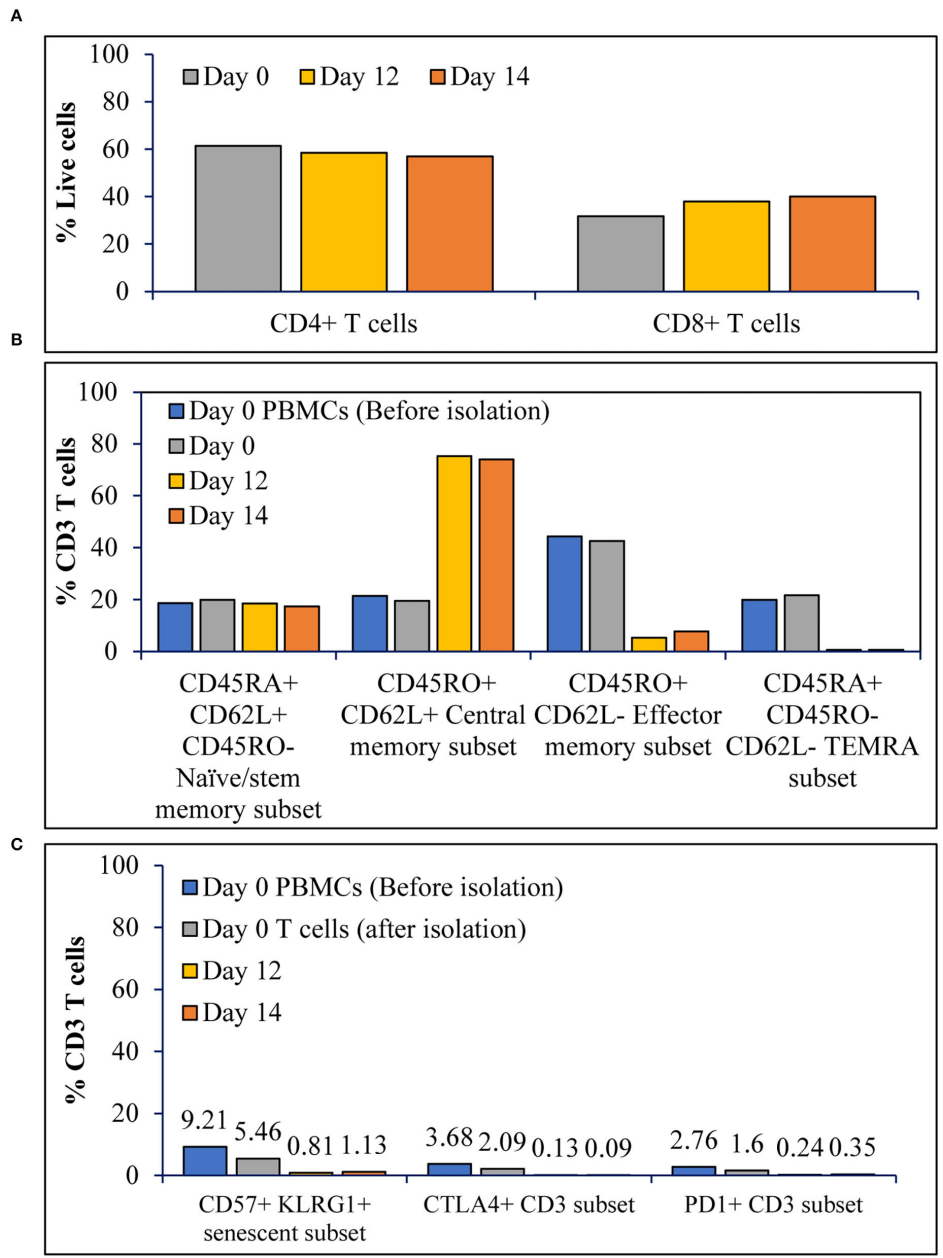
The T cell activation in STR with ATF mediated continuous perfusion results in T cells with stemness over terminal differentiation or exhaustion: **(A)** Prominent subsets of T cells before, during and after expansion; **(B)** Differentiation status of T cells before, during and after expansion; and **(C)** Senescence and exhaustion of T cells before, during and after expansion. The values represent average of 50,000 single cells per condition.

Achieving high cell expansion folds is a key for process scale-up. This attribute, however, does not hold the promise to achieve performance if cell quality is not optimal. Assessment of the CD4^+^:CD8^+^ T cell ratio for T cells expanded in the stirred tank bioreactor showed that the CD4^+^:CD8^+^ T cells ratio at inoculation was maintained during expansion with a gradual shift toward CD8^+^ T cells over time (Figure 3A). To determine the impact of expansion in stirred tank bioreactor on the phenotype of the T cells, we have the evaluated T cell phenotype during and after the expansion, in comparison to the T cell phenotype before inoculation. As shown in Figure 3B, T cell expansion resulted in ~80% central memory T cells, <10% effector memory subset, and ~15% naïve/stem memory subset. Furthermore, the expansion did not result in the accumulation of terminally differentiated (Figure 3B), senescent or exhausted T cells (Figure 3C).

### T Cell Activation and Expansion in a Stirred-Tank Bioreactor Maintains the Polyfunctionality of the Cells

**Figure 4:**
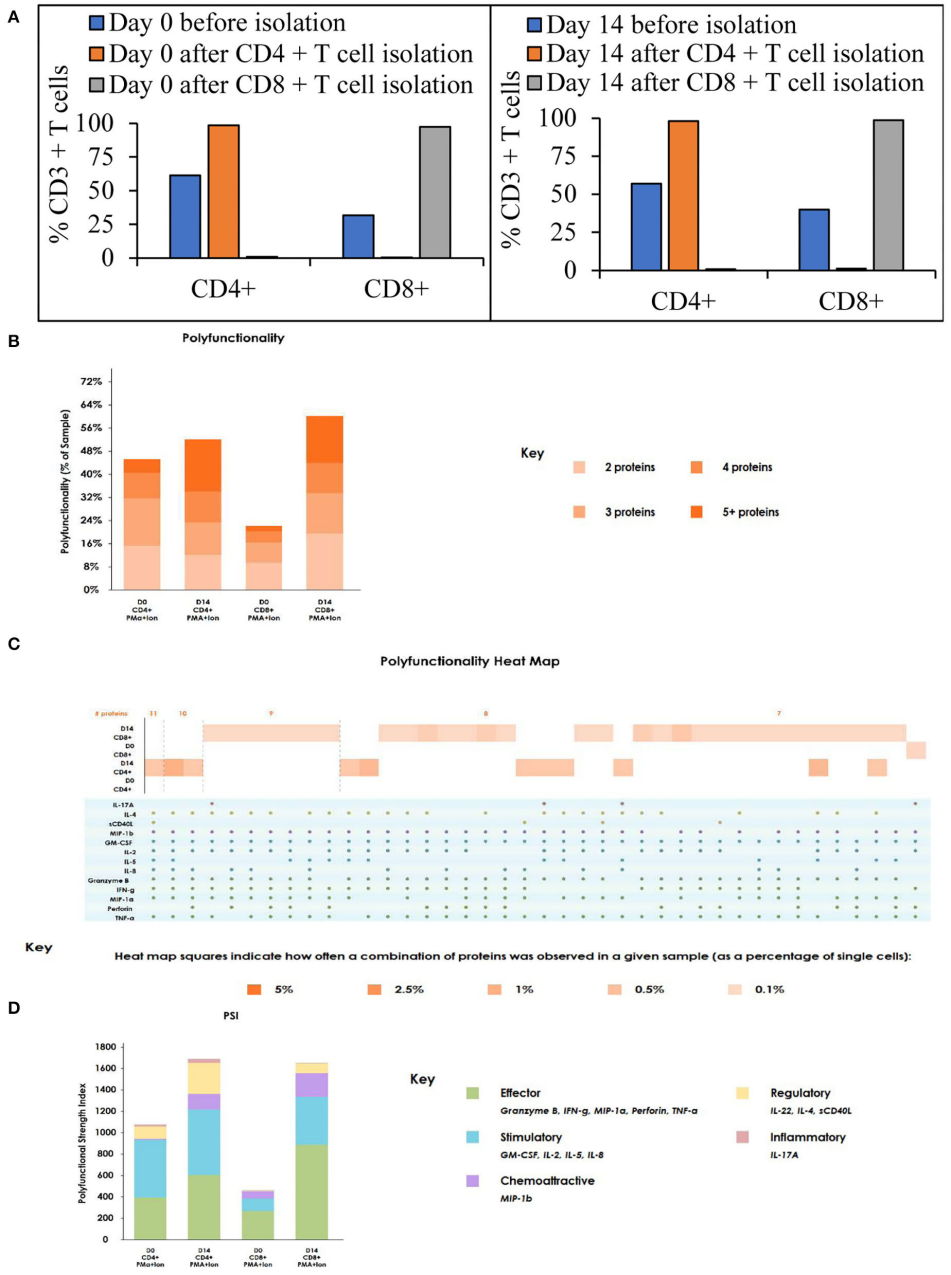
The T cell activation and expansion in stirred-tank bioreactor maintains the polyfunctionality of the T cells: **(A)** T cell subsets on Day 0 and Day 14 before and after isolation. The values represent average of 50,000 single cells per condition; **(B)** Polyfunctionality of T cells on day 0 and 14 after stimulation. The values represent average of 50,000 single cells per condition; **(C)** Heat map of polyfunctionality of T cells shown based on the number of cytokines produced; **(D)** Polyfunctional strength index of T cells on day 0 and 14 after stimulation. The values represent the average of 3,000 single cells per condition.

To assess the functional status of T cells after expansion in a stirred-tank bioreactor, we have evaluated the ability of the T cells to produce cytokines after stimulation. Isolation of CD4^+^ and CD8^+^ T cells from T cell samples obtained on day 0 and day 14 resulted in >98% pure populations (Figure 4A). The cells were stimulated, stained, and evaluated for cytokine production as described in the materials and methods. As shown in Figure 4B, the number of cells producing multiple cytokines – indicating polyfunctionality - is maintained through the expansion. In comparison to day 0, the day 14 sample showed an increased number of cells producing more than 5 cytokines. Even though at low frequency, day 14 T cells produced the highest number of cytokines, with CD4^+^ T cells producing 11 cytokines and CD8^+^ T cells producing 9 cytokines (Figure 4C). Moreover, cytokine signature-based categorization of the cell types suggests an increase in effector polyfunctional strength index whilst retaining stimulatory signature (Figure 4D).

### Efficient In-vessel Cell Depletion Using Proprietary Magnetic Technology

**Figure 5:**
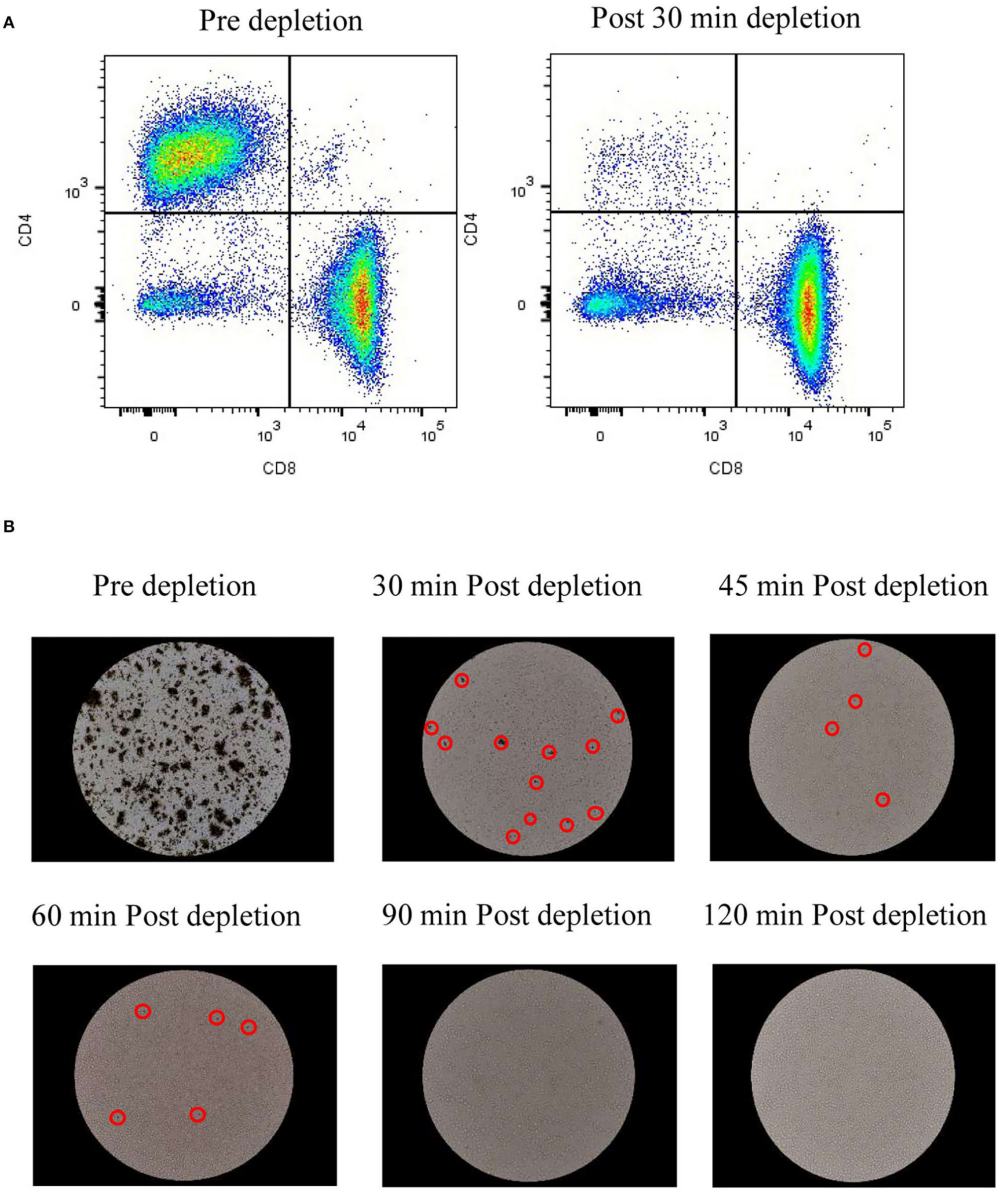
In-vessel cell depletion results in CD4^+^ T cell and bead depletion effectively: **(A)** Dot plots from flow cytometry analysis of CD8^+^ T cells vs. CD4^+^ T cells pre depletion and 30 min after depletion. The values represent average of 50,000 single cells per condition; **(B)** Images of beads in cell culture at 20X magnification before bead depletion and after incubation with magnets for specified time. The values represent reading from one experiment, unless mentioned otherwise.

As mentioned above, depletion of TCR-positive cells is needed to enable allogeneic CAR T cell-based therapy. The concentration of undesired T cells varies based on the cell editing technology and delivery platform and might result in low or high concentrations after expansion. As a proof of concept, we have evaluated the depletion of CD4^+^ T cells at low concentrations (17%) and high concentrations (52%). 30 mins of CD4^+^ T cell depletion using proprietary magnetic technology resulted in >99% depletion in lower cell concentrations and almost 97% in higher concentrations (Table 2 and Figure 5A). To assess the time required to deplete CD4^+^ T cells and beads at higher density, we have performed a time course of bead depletion. As shown in Figure 5B, the application of a magnetic field for 90 min depletes the beads, as shown by the visual bead count, post sampling. Evaluation of residual bead percentage showed to result in the presence of <.001% beads after 120 mins of bead depletion with magnets without a loss in cell viability and CD8^+^T cells (Table 3 and Figure 5B).

**Table 2:** T cell depletion efficiency. Efficiency of CD4^+^ T cell depletion for 30 min at low and high concentrations was evaluated.

**Parameter**	**Run 1**		**Run 2**	
	**Low % CD4**^**+**^ **T cells**		**High % of CD4**^**+**^ **T cells**	
	**Before CD4^**+**^ T cell depletion**	**After CD4^**+**^ T cell depletion**	**Before CD4^**+**^ T cell depletion**	**After CD4^**+**^ T cell depletion**
CD4^+^ T cell %	17%	0%	52.4%	1.64%
Depletion		>99%		96.87%
CD8^+^ T cell %	80%	97%	39.8%	85.4%

**Table 3:** Bead depletion efficiency.

**Magnetic incubation (mins)**	**Residual bead percentage**	**Decrease in viability** **(percentage)**	**Loss in CD8^**+**^ T cells (percentage)**
Pre depletion	78.370	0	
30	1.021	3.9	0
45	0.107	3.1	0
60	0.034	2.4	0
90	0.009	2.3	0
120	0.001	4.9	0

*Time course of residual bead percentage, loss in cell viability, and CD8^+^ T cells were evaluated*.

### Cell Downstream Processing Using Closed Systems Increases the Cell Density Without Loss in Cell Viability

Two mutually exclusive systems – ekko^TM^ (Millipore-Sigma) and kSep400 (Sartorius) were evaluated for cell concentration post closed to harvest. Closed-cell harvest from the STR into the harvest bag was done as described in the materials and methods. Cells from the bag were transferred in a closed manner to each of the concentration equipment. As shown in Table 4, cell concentration using ekko^TM^ resulted in a concentration of 7 fold, 0% loss in cell viability with final cell viability of 98.5%, and cell recovery of 79%. Similarly, cell concentration using kSep 400 resulted in a concentration of 7.65 fold, a 6% loss in cell viability with final cell viability of 89.4%, and cell recovery of 69%.

**Table 4:** Concentration of cells using closed systems.

**Variable**	**ekko*^*TM*^***	**kSep**
Fold concentration	7X	7.65X
Cell viability	98.5%	89.4%
Loss in viability	0%	6%
Cell recovery	79%	69%

*Fold concentration, cell viability, and recovery % were evaluated*.

## Discussion

**Figure 6:**
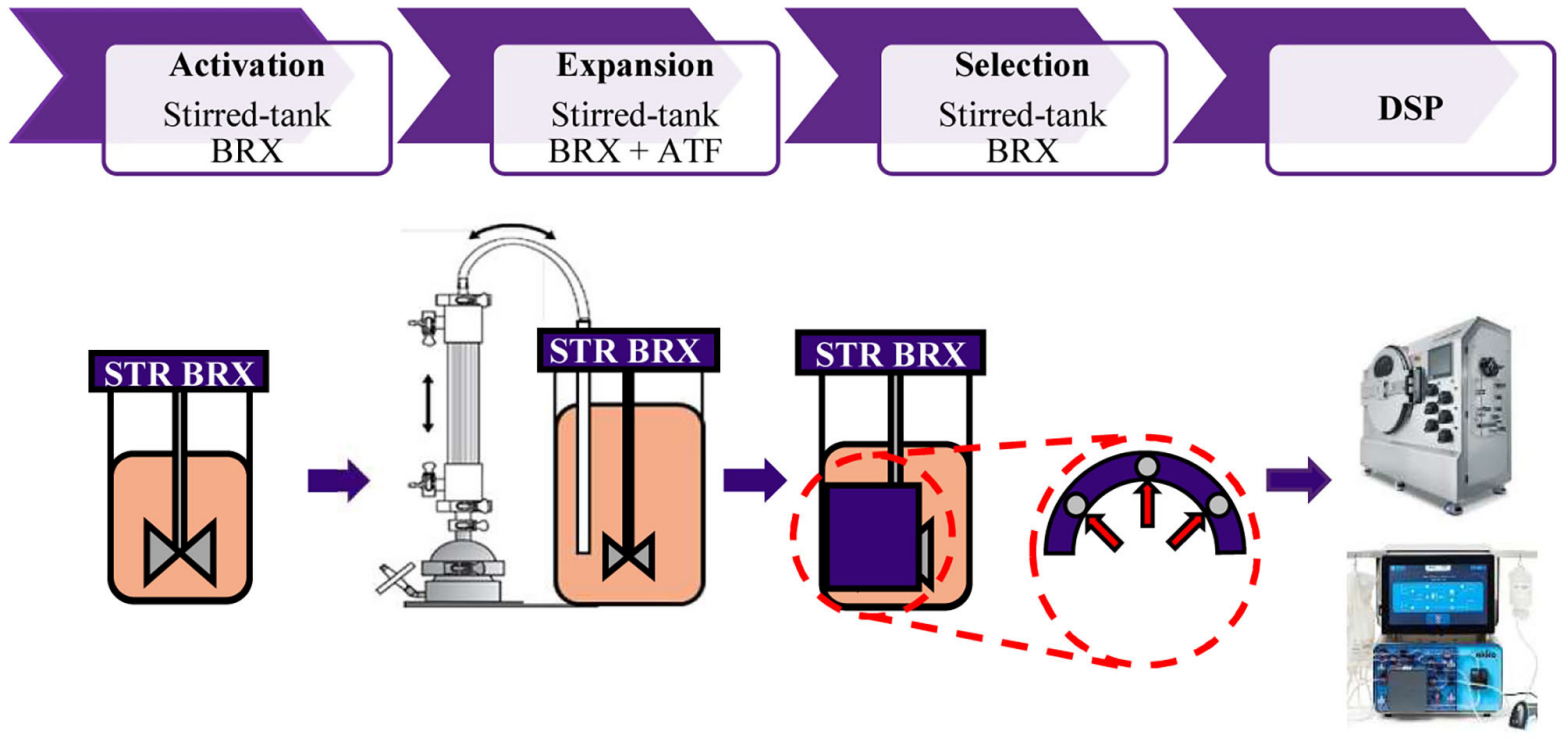
Representation of closed, GMP compatible end-to-end platform for activation, expansion, selection and downstream processing of T cells. T cell activation is performed in the STR. Cell retention and continuous media perfusion is enabled by ATF. Depletion of undesired cells is performed in the STR with proprietary magnetic technology. Cells are concentrated and formulated using automated closed systems.

Allogeneic CAR T cells can be used as ‘off-the-shelf' cell therapy and hold the promise to increase the applicability of the cell therapy product to the population on a wider scale. Current estimates suggest the need for batch sizes of 2,000 L to meet the demand for CAR T cells used for hematological and solid tumor malignancies (18). Given the large footprint, risk of contamination, variability, and poor process control in static 2D cultures (the cells are grown on flat surfaces such as culture flasks), stirred tank bioreactors to provide an excellent platform for cell expansion of T cells, offering well-characterized scale-up kinetics, in-process control and low risk of contamination. Translating 2D expansion processes into 3D expansion, in stirred tank bioreactors, was successfully shown for adherent cell types (19). However, the absence of inherent perfusion capability of STRs and the small size of T cells (5-10 mM in diameter) proved to be formidable obstacles to achieving high yields of T cells in STRs. Here, we describe a GMP-compatible, closed, and scalable platform for T cell expansion in perfusion-enabled STR. In addition, our platform offers in-unit and potentially scalable cell depletion magnetic technology, avoiding a unit operation, and lowering the risk of contamination and labor (Figure 6).

The T cell expansion in agitated conditions in spinner flasks was found to be higher, compared to static 2D culture in T25 flask (Figures 1A,B). Agitation at a constant rate of 75 RPM enabled better T cell expansion compared to agitation at a lower speed of 50 RPM during activation, followed by agitation at an increased speed of 100 RPM. Costariol et al. have shown that the expansion of primary T cells is increased with agitation speed in an automated STR (20). In agreement, we have observed that agitation at lower speeds of 25 RPM and 35 RPM did not enable T cell expansion (data not shown). A study performed by Carswell et al. has shown that T cells in spinner flasks can be expanded without detrimental effect up to 120 RPM agitation speed spinner flask (21). Maintaining the tip speed, 120 RPM in a 125 mL spinner flask is equivalent to 95 RPM in a 1-L stirred tank bioreactor. Hence, we have evaluated the T cell expansion at a lower agitation rate of 65 RPM and a higher agitation rate of 88 RPM in a 1-L stirred tank bioreactor. In agreement with the data from Carswell et al., an increased VCD of T cells was observed at higher agitation rates in an STR (Figure 1C). Cell growth accompanied by the consumption of nutrients results in the accumulation of lactate in the cell culture, which hinders efficient cell growth and quality of the final cell therapy product (22, 23). Continuous media perfusion enables a fresh supply of nutrients and the removal of harmful metabolites while retaining the cells. However, owing to the 5–10-mm diameter of T cells, cell escape, and filter fouling have been major issues related to T cell culture media perfusion. A tangential flow filtration system provides an attractive solution as a cell retention system, where the movement of fluid enables media perfusion without fouling. Furthermore, ATF provides an additional advantage of self-cleaning induced by a backflush of alternating flow (24). Repligen provides ATF devices enabling perfusion from the scales of 0.5 L to 1,000 L, serving as an attractive option for continuous media perfusion in different scales of culture in STR. As the pore size of the hollow fibers in Repligen's ATF is 0.2 mm, we have employed ATF as a cell retention device for continuous media perfusion in STR. Initial testing of ATF for cell retention and media exchange resulted in successful perfusion of 1 VVD without filter fouling and cell loss (Table 1). Post inoculation of the STR with T cells at 0.5 × 10^6^ cells/mL, T cells were activated for 3 days with CD3+28 in the presence of recombinant human IL-2. After 3 days of activation, the cells were expanded in X-VIVO^TM^ 15 media containing recombinant human IL-2. Monitoring lactate levels, we have observed a 3-fold increase in lactate levels from day 7 to day 8 (Figure 2C). This is accompanied by an increase in viable cell density to 2.1 x 10^6^ cells/mL (Figure 2B) and a drop in glucose concentration to 5.4 mM on day 8 from 6.3 mM on day 7 (Figure 2D), warranting media perfusion. Enabling media perfusion on day 8 has resulted in steady-state levels of glucose and lactate (Figures 2C,D). Furthermore, continuous media perfusion has enabled exponential growth of T cells, yielding ~35 × 10^6^ cells/mL as final VCD. In comparison, T cells from the same donor were activated and expanded in 1L G-Rex®. T cell expansion in G-Rex® resulted in a VCD of 3.4 x 10^6^ cells/mL on day 14, yielding a 28-fold expansion of T cells. A 14-day culture in STR with ATF mediated perfusion resulted in a 167-fold expansion of T cells (Figure 2B). In addition, we have observed that agitation in STR in combination with ATF-mediated cell movement did not decrease cell viability or cell expansion (Figure 2E).

Turtle et al. reported in their clinical trials that CAR T cell therapy with a 1:1 CD4:CD8 T cell ratio resulted in 93% of the patients achieving bone marrow remissions (25). In Figure 3A, we show that the CD4:CD8 T cell ratio is maintained in the STR over 14 days of expansion, suggesting a 1:1 seeding ratio would be maintained by harvest. Growing evidence suggests that the presence of a high number of naïve, stem cell memory, and central memory T cells in the final CAR T cell therapy product results in relapse-free remissions, compared to lower efficacy observed in CAR T cell products containing a higher percentage of effector memory subset (26, 27). We show that T cell expansion in the tested conditions resulted in the final T cell subtype composition containing ~80% central memory subset, ~15% naïve and stem cell memory subset, <10% effector memory, and terminal differentiated subsets (Figure 3B). This phenotype can be explained by the higher replicative potential of naïve T cells (28) and stem cell memory T cells, which replicate and differentiate upon activation (29). The higher differentiation from naïve and T memory stem cells (Tscm) combined with the higher replicative potential of central memory T cells could have yielded a higher percentage of central memory T cells. Even though final T cell subtype concentrations depend on the initial T cell composition, T cell expansion in Quantum yielded similar phenotypic results (30). Presence of senescent and exhausted T cells in the final drug product results in lower efficacy, explained by lower replication potential and dysfunctional state of the T cells, respectively (31). We show that T cell expansion in STR did not result in >5% senescent (CD57^+^ KLRG1^+^) or exhausted T cells (CTLA4^+^/PD-1^+^) (Figure 3C). Polyfunctional strength index^TM^ (PSI) of T cells was predictive of clinical responses in Acute Myeloid Leukemia and could be used as a biomarker in immunotherapy (32). PSI of the immune cells is calculated by multiplying the number of cytokines produced by each cell with the amount of each cytokine. Evaluation of PSI of CD4^+^ and CD8^+^ T cells after stimulation for 5 hours suggested an increase in the number of effectors CD4^+^ and CD8^+^ T cells from day 0 to 14. In addition to an increase in the effector signature, we have observed an increase in stimulatory signature, specifically in CD8^+^ T cells (Figure 4D). Polyfunctional T cells are capable of producing 2+ cytokines upon stimulation with antigen and are considered a vital functional characteristic (33). Evaluation of the share of cells producing multiple cytokines suggested an increase in the percentage of CD4^+^ and CD8^+^ T cells producing multiple cytokines, specifically 5+ cytokines (Figure 4B). Furthermore, ~1% of CD4^+^ T cells have produced 11 cytokines, and ~1% of CD8^+^ T cells have produced 9 cytokines upon activation (Figure 4C). As assessed by the phenotypic characterization of T cells, an increase in the central memory phenotype could be translated into an increase in the\ number of cells producing multiple cytokines (34).

Generation of allogeneic CAR T cells requires deletion of TCR alpha coding TRAC locus. Different strategies are employed to excise TRAC locus and they widely differ in their efficiency. CRISPR/Cas9 shows efficiency of 70–80%, TALEN – 60 to 80%, Zinc finger nucleases – 20 to 40% and megaTALs show an efficiency of 75% (17). Presence of TCR-positive T cells in the allogeneic cell therapy product results in GVHD (35) which could be life-threatening if involves the clonal expansion of donor T cells (36). Hence, TCR-positive T cells have to be depleted before concentration and formulation. TCR-positive T cell depletion requires harvesting T cells from STR and process through an exclusive unit, which increases the chance of contamination and incubation of cells in unoptimized conditions. We have developed a proprietary magnetic technology, which facilitates cell depletion in STR while maintaining optimized conditions. In addition, cell depletion in STR avoids the need for an additional unit operation and decreases the chance of contamination. As a proof of principle, we have depleted CD4^+^ T cells after T cell expansion. As shown in Figure 5A and Table 2, 30 min of magnetic cell depletion effectively depletes CD4^+^ T cells at lower percentages (>99%) and higher percentages (97%). As per FDA guidelines, the residual bead percentage of <0.003% in the final cell therapy product ensures the minimal effect of beads in the system. Using magnetic depletion for 120 mins, we show a decrease in the residual bead percentage to 0.001% without cell loss and a significant drop in viability (Figure 5B and Table 3). Furthermore, our proprietary magnetic technology could potentially be scaled to yield similar results at different scales.

Post depletion of undesired T cells, concentration and formulation is performed to decrease the volume, amicable for therapeutic use. We have evaluated two automated, mutually exclusive, and closed systems for cell processing and concentration. The ekko^*TM*^ performs the concentration using acoustic technology and kSep concentrates the cells by fluidized bed formation using centrifugal force. As shown in Table 4, we have concentrated the cells at least 7 fold, recovering >70% cells without a significant drop in cell viability.

To the best of our knowledge, this is the first study to describe the successful application of ATF for cell retention and media perfusion in T cell manufacturing. Collectively, our data demonstrate the capabilities of our closed, GMP compatible, end-to-end platform in expanding T cells to clinically relevant doses, purifying cell cultures, in concentration, and formulation of cell products (Figure 6).

## Conclusions

Here, we presented a closed, scalable, and automated, platform for T cell manufacturing. Continuous media perfusion leads to ~40 × 10^6^ cells/mL while maintaining nutrient and metabolite levels. Expanded cells show stemness over senescence markers. Post closed to harvest, cell concentration is enabled a closed, automated, equipment. For genetically engineered allogeneic CAR-T cells, selecting TCR-positive cells is needed. Our platform utilizes a proprietary in-vessel magnetic-based selection. This platform selectively and efficiently depletes undesired cell populations, avoiding the need for cell transfer into an additional unit of operation. This enables maintaining the cells under optimal culture conditions during the selection step and reduces contamination risk. Moreover, our proprietary in-vessel selection technology is highly scalable, and prepared to meet future clinical demand for a large number of allogeneic CAR-T cells.

## Data Availability Statement

Data may be available upon request at Lonza's discretion. Requests to access these datasets should be directed to Lonza, pharma@lonza.com.

## Author Contributions

HG, NU, and AS designed the various study parts, conducted experiments, and analyzed the data. AN contributed to the execution of the STR BRX runs. HG, NU, and AS have contributed to methodology development. YL, SR, and IF contributed to the conception and design of the study and gave intellectual input throughout the project and to the analysis of the data. HG wrote the manuscript. IF and AN provided critical revision to the manuscript. All authors approved the final manuscript.

## Conflict of Interest

All authors are current or previous employees of Lonza, a pharmaceutical company that develops and sells a wide range of products, including cell-based products for research and pharmaceutical use. Lonza may derive benefit from the sale of a product derived from this research. However, this does not alter our adherence to all the IJMS policies on sharing data and materials.

## Publisher's Note

All claims expressed in this article are solely those of the authors and do not necessarily represent those of their affiliated organizations, or those of the publisher, the editors and the reviewers. Any product that may be evaluated in this article, or claim that may be made by its manufacturer, is not guaranteed or endorsed by the publisher.
